# Up-to-date survival curves of children with cancer by period analysis

**DOI:** 10.1038/sj.bjc.6600947

**Published:** 2003-05-27

**Authors:** H Brenner

**Affiliations:** 1 Department of Epidemiology, German Centre for Research on Ageing, Bergheimer Str. 20, D-69115 Heidelberg, Germany

**Keywords:** children, cancer registries, statistical methods, survival

## Abstract

Survival rates of children with cancer have strongly improved during the past decades, but much of this improvement has been disclosed with substantial delay by traditional methods of survival analysis, which reflect survival experience of patients diagnosed many years ago. In this paper, the use of a new method of survival analysis, denoted period analysis, for providing more up-to-date estimates of 10-year survival curves of children with cancer is empirically evaluated using data of the Surveillance, Epidemiology, and End Results Program of the United States National Cancer Institute. It is shown that period analysis provides much more up-to-date estimates of survival curves than traditional cohort-based survival analysis indeed, at least as long as there is ongoing improvement in survival rates over time, as it seems to be the case for many forms of childhood cancer. The most recent 10-year period survival estimates indicate that survival rates of children with cancer achieved by the end of the 20th century are substantially higher than previously available survival statistics have suggested. Application of period analysis may be particularly useful in the field of childhood cancer as it may help to prevent patients, their families and clinicians from being burdened by outdated, often too pessimistic survival expectations.

Although childhood cancer is rare, accounting for about 1% of all cancer cases only (Little, 1999), the associated burden for the patients and their families is immense. Fortunately, prognosis for children with cancer has greatly improved over the past decades, principally owing to more effective chemotherapy (La Vecchia *et al*, 1998; Ries *et al*, 1999).

The most commonly reported measures of prognosis of children with cancer are 5-year survival rates (e.g. Gatta *et al*, 2002). Although most deaths among children with cancer occur during the first 5 years following diagnosis, the proportion of late deaths occurring beyond the fifth year following diagnosis is not negligible for many forms of childhood cancers (Mertens *et al*, 2001; Möller *et al*, 2001). Therefore, more long-term survival statistics are of particular interest for childhood cancer.

However, traditional estimates of long-term survival rates (Cutler and Ederer, 1958; Kaplan and Meier, 1958), which pertain to cohorts of patients diagnosed many years ago, may be overly pessimistic in case of recent improvements in prognosis. A few years ago, a new method of survival analysis, denoted period analysis, has been introduced to provide more up-to-date estimates of long-term survival rates (Brenner and Gefeller, 1996,^1997^). For adulthood cancers, performance of period analysis has meanwhile undergone extensive empirical evaluation, which demonstrated that this method provides much more up-to-date estimates of long-term survival rates than the traditional methods of survival analysis indeed (Brenner and Hakulinen, 2002a,2002b,2002c; Brenner *et al*, 2002b). However, no systematic evaluation has been carried out for childhood cancers, and, with few exceptions (Brenner *et al*, 2001; Burkhardt-Hammer *et al*, 2002), the method has not been used for monitoring progress in childhood cancer so far.

The aim of this analysis was to provide an empirical evaluation of the performance of period analysis for deriving up-to-date estimates of 10-year survival curves of children with cancer.

## MATERIALS AND METHODS

All data presented in this paper are derived from the 1973–1999 public use database of the Surveillance, Epidemiology, and End Results (SEER) Program (2002) of the United States National Cancer Institute. Although the SEER Program is not a true nationwide population-based cancer registry scheme, it is the most authoritative source of information on cancer incidence and survival in the US, and it is considered as the standard for quality among cancer registries around the world. Quality control has been an integral part of SEER since its inception. Every year, studies are conducted in the SEER areas to evaluate the quality and completeness of the data being reported (SEER's standard for case ascertainment is 98%).

Data included in the 1973–1999 SEER database are from population-based cancer registries in Connecticut, New Mexico, Utah, Iowa, Hawaii, Atlanta, Detroit, Seattle-Puget Sound and San Francisco-Oakland, which together cover a population of about 24 million people. In this analysis, patients with a first diagnosis of cancer below age 15 years between 1975 and 1999, who have been followed for vital status until the end of 1999 are included. Patients with missing information on month or year of diagnosis (0.3%) or survival time (0.6%) were excluded, as were patients whose cancer was reported by death certificate only (0.2%) or autopsy only (0.4%). Data are presented for all races and both sexes combined. Specific analyses are shown for various age groups (0–4, 5–9, 10–14 years) and the four most common diagnostic groups according to the International Classification of Childhood Cancer: leukaemias, lymphomas, central nervous system and miscellaneous intracranial and intraspinal neoplasms, and sympathetic nervous system tumours. The focus of this analysis is on 10-year survival rates rather than the more commonly reported 5-year survival rates.

**Figure 1:**
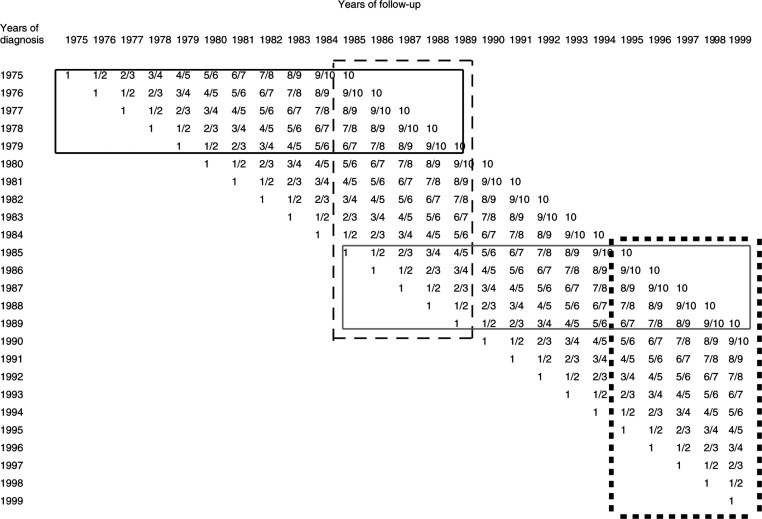
Database for the 10-year survival curves actually observed for patients diagnosed in 1985–1989 (solid grey frame), and for the most up-to-date estimates of 10-year survival curves available in 1985–1989 by period analysis (dashed black frame) or cohort analysis (solid black frame). The black squares frame indicates the database for the most up-to-date period estimates of 10-year survival curves available in 1995–1999. The numbers within the cells indicate the years of follow-up since diagnosis.

The principle of period analysis has been described in detail elsewhere (Brenner and Gefeller, 1996,^1997^). Briefly, period estimates of survival for a recent time period are obtained by left truncation of observations at the beginning of that period in addition to right censoring at its end. The approach used for the empirical evaluation is illustrated in Figure 1

: 10-year survival curves actually observed for children diagnosed with cancer between 1985 and 1989 (the most recent cohort of children for whom 10-year follow-up was complete at the time of this analysis, solid grey frame in Figure 1) are compared with the most up-to-date estimates of 10-year survival curves that might have been available in 1985–1989 (i.e. at the time of diagnosis of these children) using either period analysis or traditional cohort analysis. For simplicity, any delay in cancer registration, mortality follow-up, and data analysis are neglected. The 10-year survival curves available in 1985–1989 by traditional cohort analysis would have pertained to survival experience in 1975–1989 of patients diagnosed in 1975–1979 (solid black frame). By contrast, 10-year survival curves obtained by period analysis would have exclusively reflected survival experience in 1985–1989. This analysis would have included patients diagnosed in 1975–1989, but all observations would have been left truncated at the beginning of 1985 and right censored at the end of 1989 (dashed black frame). With that approach, survival experience during the first year following diagnosis is provided by patients diagnosed between 1984 and 1989, survival experience in the second year following diagnosis is provided by patients diagnosed between 1983 and 1988 and so on, until survival experience during the 10th year after diagnosis, which is provided by patients diagnosed between 1975 and 1980.

In addition, analogously derived 10-year period survival curves for the most recent period (1995–1999) for which data were available at the time of this analysis are also given (black squares frame).

All analyses were performed with the SAS software package using a publicly available macro for both cohort and period analysis, which is described in detail elsewhere (Brenner *et al*, 2002a). Since mortality from competing causes of death is almost negligible for children, and relative survival rates are essentially the same as absolute survival rates for this age group, only absolute survival rates are presented in this paper. Standard errors of survival rates were calculated according to Greenwood's (1926) method.

## RESULTS

Table 1

**Table 1:** Number of children diagnosed with cancer below age 15 years between 1975 and 1999 included in this analysis

**Overall**	**18 100 (100.0%)**
*By age at diagnosis (years)*	
0–4	8018 (44.3%)
5–9	5029 (27.8%)
10–14	5053 (27.9%)
	
*By the most common diagnostic groups*	
Leukaemias	5664 (31.3%)
Lymphomas	1825 (10.1%)
Central nervous system and miscellaneous intracranial and intraspinal neoplasms	3776 (20.9%)
Sympathetic nervous system tumours	1449 (8.0%)

shows the numbers of children, by age and diagnostic groups, who were included in this analysis. Overall, 18 100 children were included. Almost half of the children were less than 5 years old at the time of diagnosis, about a quarter of the children were in each of the other two age groups (5–9 and 10–14 years). The most common diagnostic group was leukaemia, which accounted for 31% of all childhood neoplasms (acute lymphocytic leukaemia: 24%, other leukaemia: 7%), followed by central nervous system neoplasms (overall: 21%, astrocytoma: 10%, primitive neuroectodermal tumours: 5%, others: 6%), lymphomas (overall: 10%, Hodgkin's lymphoma: 4%, other lymphomas: 6%), and sympathetic nervous system tumours (8%). The latter group overwhelmingly consists of neuroblastomas and ganglioneuroblastomas (7.8%). Each of the four diagnostic groups specifically addressed in this analysis included more than 1400 children.

Figure 2

**Figure 2:**
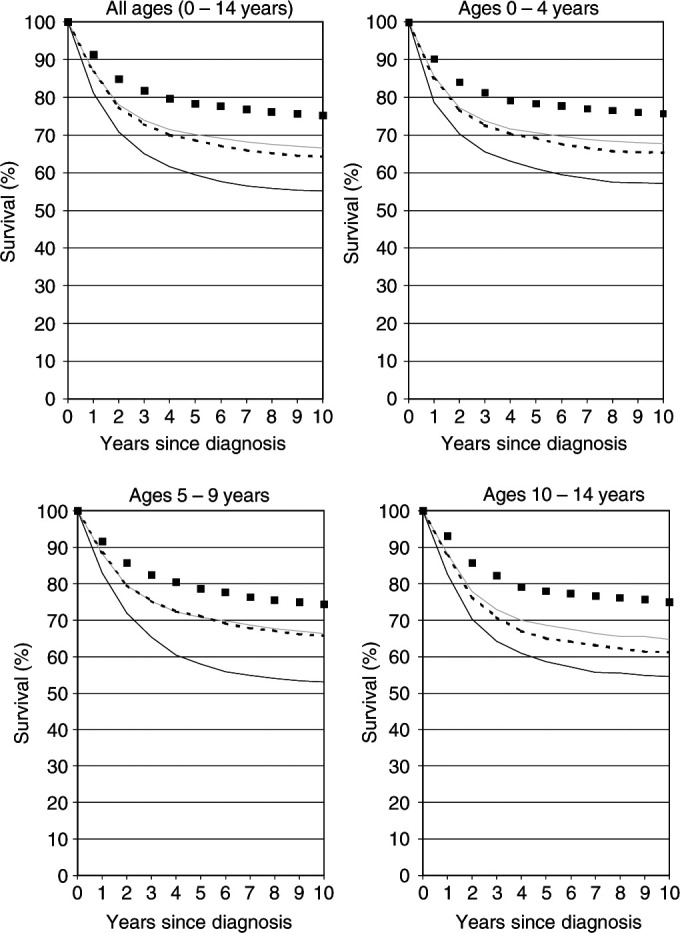
Observed 10-year survival curves of children diagnosed with any form of cancer at various ages in 1985–1989 (grey lines) compared with the most up-to-date survival curves available in 1985–1989 by period analysis (dashed black lines) and cohort analysis (solid black lines). The black squares indicate the period survival curves for the 1995–1999 period.

shows the observed 10-year survival curves of children diagnosed with any form of cancer at various ages in 1985–1989 (grey lines) compared with the most up-to-date survival curves available in 1985–1989 by period analysis (dashed black lines) and cohort analysis (solid black lines). Overall, and within each single age group, the 10-year survival curves available by period analysis in 1985–1989 would have come much closer to the 10-year survival curves later observed for patients diagnosed in that time period than the corresponding 10-year survival curves available by cohort analysis. Whereas cohort estimates of 10-year survival available in 1985–1989 would have been between 10.2 and 13.5 percent units lower than the 10-year survival rates later observed for children diagnosed in that period, the discrepancy would have been between 0.6 and 3.5 percent units only for the period estimates. Nevertheless, even the period estimates tended to be lower than the 10-year survival rates later observed for newly diagnosed patients in all cases.

**Figure 3:**
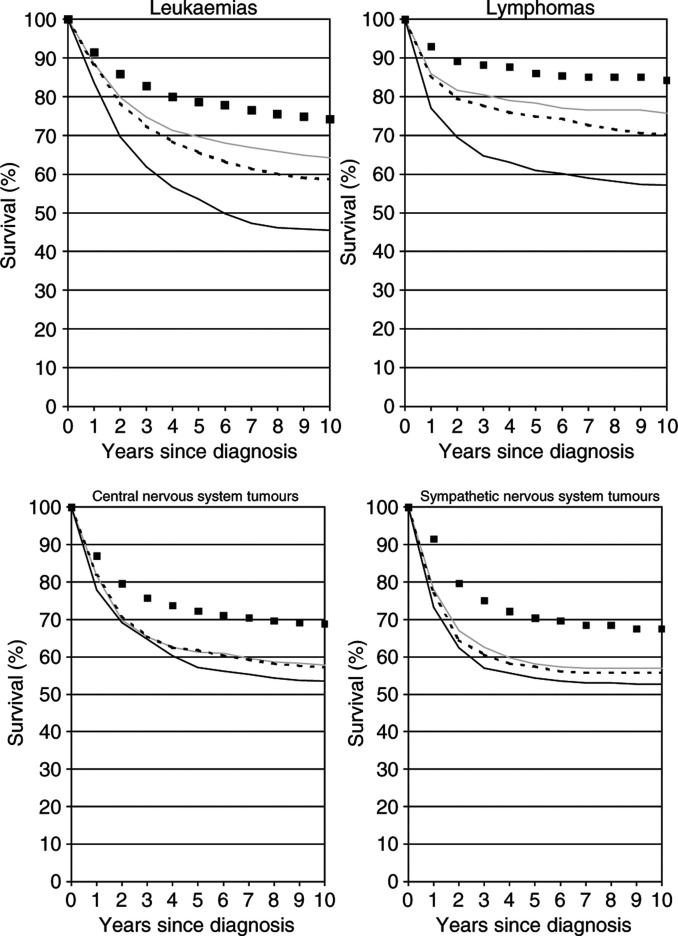
Observed 10-year survival curves of children diagnosed with various forms of cancer in 1985–1989 (grey lines) compared with the most up-to-date survival curves available in 1985–1989 by period analysis (dashed black lines) and cohort analysis (solid black lines). The black squares indicate the period survival curves for the 1995–1999 period.

The increase in 10-year survival between patients diagnosed in 1975–1979 and 1985–1989 was particularly strong for children with leukaemias and lymphomas (+18.9 and +18.7 percent units, respectively) (see Figure 3

, upper two graphs). Accordingly, children diagnosed with these neoplasms in the late 1980s were confronted with much too pessimistic 10-year survival statistics obtained by traditional cohort analysis, which equal the 10-year survival rates of patients diagnosed 10 years earlier. Period estimates of 10-year survival that could have been derived in 1985–1989 would also have been somewhat too pessimistic, but much less so than the traditional cohort estimates. Despite differences in absolute levels of survival rates, this pattern was seen in additional analyses for each of the major subgroups (acute lymphocytic leukaemia, other leukaemias, Hodgkin's lymphoma, and other lymphomas; data not shown).

For children diagnosed with neoplasms of the central and sympathetic nervous system in 1985–1989, 10-year survival rates were somewhat lower (below 60%) (see Figure 3, lower two graphs). The latest cohort estimates of 10-year survival available at the time of their diagnosis would again have been somewhat too pessimistic, although the differences were not as large as for the other diagnostic groups. On the other hand, the latest period estimates available in those years would almost perfectly have predicted the 10-year survival curves observed 10 years later for these children. Additional analysis revealed that this was true for each of the major subgroups (astrocytoma, primitive neuroectodermal tumours, other central nervous system tumours, neuroblastoma, and ganglioneuroblastoma) within these diagnostic groups (data not shown).

Of course, it would be of great interest to know the 10-year survival rates to be expected by children diagnosed in more recent years. A definitive answer to this question will only be available 10 years from now. However, the empirical evaluation described above suggests that the period estimates of 10-year survival curves for the 1995–1999 period, which are depicted by black squares in Figure 2 and Figure 3, do come quite close to the 10-year survival curves of patients diagnosed in that period. Although these survival curves may actually still be too pessimistic, they suggest that there has been substantial further recent improvement in survival rates in all age groups and for all diagnostic groups considered in this analysis. The 1995–1999 period estimate of 10-year survival for all ages and all forms of childhood cancer combined is 75.2%, 8.7 percent units higher than the 10-year survival rate for the 1985–1989 cohort (grey solid curve), which is the latest cohort estimate that can be derived from the current data base.

In Figure 2 and Figure 3, only point estimates of survival rates are shown. Of course, it is also important to take their standard errors into account. Standard errors were generally of similar magnitude for period and cohort estimates. The standard errors for the different 10-year survival rates ranged from 0.7 to 0.9% for all cancers combined, from 1.1 to 1.4, 1.4 to 1.7, and 1.4 to 1.7% for age groups 0–4, 5–9 and 10–14 years, respectively, and from 1.3 to 1.7, 2.0 to 2.6, 1.7 to 2.0, and 2.8 to 3.2% for leukaemias, lymphomas, central and sympathetic nervous system neoplasms, respectively.

## DISCUSSION

To my knowledge, this is the first systematic evaluation of the use of period analysis for deriving up-to-date long-term survival curves of children with cancer. It could be shown that period analysis provides much more up-to-date estimates of survival curves than traditional cohort-based survival analysis indeed, at least as long as there is ongoing improvement in survival rates over time, as it seems to be the case for each of the most common forms of childhood cancer. These results are consistent with the results of previous empirical evaluations of the method's performance in the analysis of cancer survival among adults (Brenner and Hakulinen, 2002a,2002b,2002c; Brenner *et al*, 2002b). However, because improvement in prognosis has been stronger for most childhood cancers than for cancers among adults, the advantage of period analysis over traditional survival analysis with respect to derivation of up-to-date survival rates appears to be even larger for childhood cancer.

Despite being more up-to-date than the traditional cohort-based survival curves, even the period survival curves tended to be somewhat too pessimistic in all cases (albeit much less so than the traditional cohort-based survival curves). As expected from theory, this particularly applied to those neoplasms for which the most rapid increase in survival rates was observed (Brenner and Gefeller, 1996,^1997^), namely leukaemias and lymphomas. In theory, period estimates of long-term survival curves may also become (transiently) overoptimistic, if advancements in early detection or therapy do not increase chances of cure, but merely postpone cancer deaths. In fact, this theoretical possibility has been repeatedly forwarded as a caveat against the use of period analysis, and it has been suggested to be of particular concern for childhood cancers, as a consequence of possible late adverse treatment effects. However, studies have shown that modern treatment of childhood cancers tended to reduce rather than increase occurrence of late cancer deaths (in addition to reducing occurrence of early cancer deaths) (Robertson *et al*, 1994; Hudson *et al*, 1997; Möller *et al*, 2001). In agreement with these findings, our empirical evaluation suggests that the theoretical possibility of overoptimistic survival estimates by period analysis appears to be as irrelevant in practice as it is for adulthood cancers (Brenner and Hakulinen, 2002a,2002b,2002c; Brenner *et al*, 2002b). In the contrary, the issue to be concerned about is that even the most up-to-date period estimates of long-term cancer patient survival available at a given time are usually too pessimistic if there is ongoing improvement in prognosis (as it seems to be the case for most childhood cancers). Therefore, even the most up-to-date period estimates should usually be regarded as conservative (possibly too low) estimates of long-term survival rates achieved at a given time period and to be expected by newly diagnosed patients in that period.

In the interpretation of the results, the following limitations should be kept in mind. Although, overall, a very large number of children were included in this analysis, sample size limitations hindered a more detailed analysis for the less common diagnostic groups or for single forms of childhood cancer. Restriction of separate analyses to the most common diagnostic groups, along with presentation of results by 5-year calendar intervals rather than single calendar years ensured that the largest standard error for any of the presented 10-year survival estimates was below 3.3% in all cases, and within the range from 1 to 2.5% for the majority of the presented 10-year survival estimates. There is also no obvious reason, why the performance of period analysis should be different for those diagnostic groups for which no separate analysis was carried out because of sample size limitations. An additional analysis for all those diagnostic groups combined yielded the same overall patterns for the performance of period analysis compared to cohort analysis illustrated for the more common diagnostic groups in this paper (data not shown).

Although this paper addressed 10-year survival rates rather than the more commonly reported 5-year survival rates, even longer-term survival rates, such as 20- or 30-year survival rates would be of interest for children with cancer, given that the occurrence of late deaths is not negligible for these children, and given the much longer life expectancy children with cancer would have in the absence of cancer compared to adult cancer patients. However, an empirical evaluation of the performance of period analysis for such long-term survival rates would require much longer time series of cancer registration than the 27-year time series meanwhile accumulated in the SEER program. For adulthood cancers, such evaluations have been carried out using the almost 50-years-long time series of the Finnish Cancer Registry. These analyses suggested, that the advantage of period analysis over traditional survival analysis for deriving up-to-date survival estimates is even larger for these longer-term survival rates (Brenner and Hakulinen, 2002c).

Presentation of results in this paper focused on comparison of results obtained by period analysis with those obtained by frequently used cohort analysis, in which only patients who have been under examination for the entire follow-up interval of interest are included. Other conventional analyses have also included patients who were not seen for the entire follow-up interval of interest and whose survival time was censored at the closing date of follow-up (unless they died or were lost before that date) (e.g. Stiller and Eatcock, 1994,^1999^; Mott, 1995). Additional analysis of survival rates by the so-called ‘complete analysis’ (including all patients diagnosed prior to the closing date of follow-up, see Brenner and Gefeller, 1997) confirmed previous observations (Brenner and Hakulinen, 2002a; Brenner *et al*, 2002b) that, although this type of analysis provides more up-to-date (and precise) long-term survival estimates than conventional cohort analysis, these estimates are, nevertheless, still much less up-to-date than those obtained by period analysis (data not shown).

In conclusion, this empirical evaluation suggests that the period analysis approach, which has long been used in other fields of sciences, such as demography, is at least as useful for deriving more up-to-date estimates of long-term survival for childhood cancer as it is for adulthood cancers. The 10-year period survival estimates for the 1995–1999 period also indicate that survival rates of children with cancer achieved by the end of the 20th century are higher than previously available survival statistics have suggested. For example, the 10-year survival period estimate for the 1995–1999 period for all forms of cancer combined (75%) is even higher than the recently published cohort estimate of 5-year survival of 70% (pertaining to the 1985–1989 cohort of children in the same data base) (Gatta *et al*, 2002). Application of period analysis may be particularly useful in the field of childhood cancer as it may help to prevent patients, their families and clinicians from being burdened by outdated, often too pessimistic survival expectations.
